# Absence of tmRNA Has a Protective Effect against Fluoroquinolones in *Streptococcus pneumoniae*

**DOI:** 10.3389/fmicb.2016.02164

**Published:** 2017-01-10

**Authors:** Liliana Brito, Joana Wilton, María J. Ferrándiz, Alicia Gómez-Sanz, Adela G. de la Campa, Mónica Amblar

**Affiliations:** ^1^Unidad de Patología Molecular del Neumococo, Centro Nacional de Microbiología, Instituto de Salud Carlos IIIMadrid, Spain; ^2^Unidad de Genética Bacteriana, Centro Nacional de Microbiología, Instituto de Salud Carlos IIIMadrid, Spain; ^3^Presidencia, Consejo Superior de Investigaciones CientíficasMadrid, Spain

**Keywords:** *Streptococcus pneumoniae*, tmRNA, *trans*-translation, stress adaptation, antibiotic resistance, fluoroquinolones, chromosomal fragmentation, reactive oxygen species

## Abstract

The transfer messenger RNA (tmRNA), encoded by the *ssrA* gene, is a small non-coding RNA involved in *trans*-translation that contributes to the recycling of ribosomes stalled on aberrant mRNAs. In most bacteria, its inactivation has been related to a decreased ability to respond to and recover from a variety of stress conditions. In this report, we investigated the role of tmRNA in stress adaptation in the human pathogen *Streptococcus pneumoniae*. We constructed a tmRNA deletion mutant and analyzed its response to several lethal stresses. The Δ*ssrA* strain grew slower than the wild type, indicating that, although not essential, tmRNA is important for normal pneumococcal growth. Moreover, deletion of tmRNA increased susceptibility to UV irradiation, to exogenous hydrogen peroxide and to antibiotics that inhibit protein synthesis and transcription. However, the Δ*ssrA* strain was more resistant to fluoroquinolones, showing twofold higher MIC values and up to 1000-fold higher survival rates than the wild type. Deletion of SmpB, the other partner in *trans*-translation, also reduced survival to levofloxacin in a similar extent. Accumulation of intracellular reactive oxygen species associated to moxifloxacin and levofloxacin treatment was also highly reduced (∼100-fold). Nevertheless, the Δ*ssrA* strain showed higher intracellular accumulation of ethidium bromide and levofloxacin than the wild type, suggesting that tmRNA deficiency protects pneumococcal cells from fluoroquinolone-mediated killing. In fact, analysis of chromosome integrity revealed that deletion of tmRNA prevented the fragmentation of the chromosome associated to levofloxacin treatment. Moreover, such protective effect appears to relay mainly on inhibition of protein synthesis, since a similar effect was observed with antibiotics that inhibit that process. The emergence and spread of drug-resistant pneumococci is a matter of concern and these results contribute to a better comprehension of the mechanisms underlying fluoroquinolones action.

## Introduction

The transfer messenger RNA (tmRNA) is a ubiquitous specialized small non-coding RNA encoded by the *ssrA* gene that functions as both a tRNA and an mRNA. It works together with the SmpB protein in the *trans-*translation system, a quality control pathway that rescues ribosomes stalled on non-stop mRNAs (Keiler et al., 1996; Karzai et al., 1999, 2000). During *trans*-translation, the tmRNA-SmpB complex shifts the translation of the nascent peptide from the aberrant mRNA to the tmRNA-coding sequence, allowing resumption of translation while targeting the peptide for degradation and recycling ribosomes (Giudice et al., 2014; Shimizu, 2014). Accumulation of stalled ribosomes is toxic and they need to be rescued, otherwise, the cell would rapidly be depleted of translational ribosomes and protein synthesis would come to halt. Some bacteria have alternative backup systems that use either ArfA or ArfB factors to recognize no-stop complexes, promote hydrolysis of the peptidyl-tRNA and release the stalled ribosome (Chadani et al., 2010, 2011; Schaub et al., 2012; Keiler and Feaga, 2014). Impairment of *trans*-translation leads to a wide variety of phenotypes, likely influenced by the status of ArfA and/or ArfB. These phenotypes range from very subtle growth defects to lethality, but the most common are associated to defects in pathogenicity and stress-adaptation (Oh and Apirion, 1991; Komine et al., 1994; Julio et al., 2000; Muto et al., 2000; Okan et al., 2006; Li et al., 2008; Barends et al., 2010; Nichols et al., 2011; Mann et al., 2012; Svetlanov et al., 2012; Mu et al., 2013), or to increased sensitivity to antibiotics targeting translation (de la Cruz and Vioque, 2001; Abo et al., 2002; Vioque and de la Cruz, 2003; Luidalepp et al., 2005; Okan et al., 2006; Nichols et al., 2011; Svetlanov et al., 2012). In addition to quality control pathways, some genetic regulatory circuits use *trans*-translation to control gene expression, and diverse bacteria require *trans*-translation when they execute large changes in their genetic programs (Abo et al., 2000; Christensen and Gerdes, 2003; Christensen et al., 2003; Keiler and Shapiro, 2003; Ranquet and Gottesman, 2007; Keiler, 2008; Kobayashi et al., 2008). Therefore, the contribution of tmRNA and the *trans*-translation mechanism to cell survival differs among bacteria, and key questions regarding their utility remain unanswered.

We wanted to investigate the role of the tmRNA in the human pathogen *Streptococcus pneumoniae*. This bacterium is responsible for a wide spectrum of human diseases, ranging from mild otitis media to more severe infections such as meningitis, sepsis, or endocarditis (WHO, 2007)^1^. It is the most common bacterial cause of community-acquired pneumonia and the leading cause of vaccine-preventable deaths in children <5 years old (O’Brien et al., 2009). Treatment of pneumococcal diseases is hampered by the emergence and spread of drug-resistant strains to traditionally effective agents, including beta-lactam antibiotics (Jacobs et al., 2003) and macrolides (Sahm et al., 2001). Fluoroquinolones (FQs), such as levofloxacin (LVX) and moxifloxacin (MOX), are currently used for the treatment of pneumococcal pneumonia in adult patients. These antibiotics inhibit type II DNA topoisomerases, ubiquitous enzymes that manage DNA topology and solve topological problems associated with DNA replication, transcription, and recombination (Champoux, 2001). It has been proposed that several FQs require ongoing protein synthesis to cause cell death, and that protein synthesis inhibitors may protect from chromosome fragmentation (Chen et al., 1996; Malik et al., 2006, 2007). In addition, reactive oxygen species (ROS) such as superoxide, hydrogen peroxide (H_2_O_2_) and hydroxyl radical, contribute to FQ-mediated killing (Dwyer et al., 2007, 2014; Kohanski et al., 2007; Wang and Zhao, 2009; Wang et al., 2010). In *S. pneumoniae*, the mechanisms leading to ROS accumulation mediated by LVX and MOX have been recently elucidated (Ferrándiz and de la Campa, 2014; Ferrándiz et al., 2016). Both FQs induce global transcriptional responses that, although through different pathways, ultimately stimulate the Fenton reaction, increasing ROS accumulation and contributing to cell death.

Expression of tmRNA in the pneumococcus was recently demonstrated (Kumar et al., 2010; Acebo et al., 2012; Mann et al., 2012; Wilton et al., 2015) and, although no functional studies have been reported so far, its deficiency has strong effects in pathogenesis. In fact, ssrA inactivation reduced the ability to adhere and to invade endothelial cells, reduced the fitness and competitive index in lungs and causes attenuation in invasive diseases upon intranasal challenge (Mann et al., 2012). Regarding SmpB, its partner in *trans*-translation, it has been demonstrated that expression of the pneumococcal SmpB protein is induced under cold-shock and that its levels are regulated by RNase R, an exoribonuclease involved in degradation of faulty mRNAs released from stalled ribosomes during *trans-*translation (Richards et al., 2006; Moreira et al., 2012). However, a direct link between *trans*-translation and survival to stress or antibiotic susceptibility/resistance, have never been established in *S. pneumoniae*.

The aim of the present study was to investigate the role of tmRNA in adaptation of *S. pneumoniae* to environmental conditions and antibiotic stress. We examined how the absence of tmRNA affects survival to several lethal environmental conditions and the activity of chemically unrelated antibiotics, some of which are commonly used in clinical practice. We showed that tmRNA deficiency had a detrimental effect on growth and was more sensitive to UV, H_2_O_2_ and to a variety of antibiotics. However, deletion of tmRNA highly increased bacterial survival against FQs, decreased accumulation of intracellular ROS and reduced chromosomal fragmentation. This is the first study reporting a higher FQ-resistance phenotype associated to tmRNA deficiency, which has been generally considered as a stress-adaptation RNA.

## Materials and Methods

### Bacterial Strains, Plasmids, Growth Conditions, and Transformation

*Streptococcus pneumoniae* strains and plasmids used in this study are described in **Table 1**. Pneumococci were grown as static cultures either in Todd-Hewitt medium supplemented with 0.5% of yeast extract (THY), or in a casein hydrolase-based medium (AGCH) supplemented with 0.3% sucrose and 0.2% of yeast extract (A+SY). All constructs and cloning experiments were carried out in the pneumococcal R6 strain. TIGR4 cells were transformed as previously described (Acebo et al., 2012) and plated onto blood agar plates. R6 cells were transformed as described previously (Lacks et al., 1986) and transformants were plated on A+SY media plates containing 1% agar. Incubations were performed at 37°C in a 5% CO_2_ atmosphere. The pROM plasmid was constructed by deleting the 173 bp fragment containing the maltose-inducible promoter (PM) of pLS1ROM (Ruiz-Masó et al., 2012). For this purpose, the whole plasmid (excluding PM) was amplified through reverse PCR using pROM-F-Xba2 and pROM-R-Xba2 primers (**Table 2**). The fragment was then digested with *Xba*I and further ligated to obtain pROM. The pROM-TM plasmid was obtained by cloning a 531 bp chromosomal region containing the entire tmRNA encoding gene *ssrA* gene (including its own promoter) into pROM. This fragment was amplified by PCR from TIGR4 chromosomal DNA using Expand High Fidelity (Roche). Primers used were tmRNACj-F and tmRNACj-R (**Table 2**), which contained the *BamH*I and *Hind*III restriction sites, respectively. The PCR product was cloned into pROM vector making use of the *BamH*I and *Hind*III restriction sites, thus obtaining the pROM-TM plasmid, which was then transformed into R6 competent cells. Transformants were selected using 1 μg/ml of erythromycin and cloning was verified by DNA sequencing. Expression of tmRNA in *trans* was confirmed by Northern-blot as previously described (Acebo et al., 2012).

**Table 1:** Bacterial strains and plasmids used in this study.

Bacterial strain	Description	Source
TIGR4	Capsular type 4 clinical isolate strain TIGR4	Tettelin et al., 2001
TIGR4*ΔssrA*	TIGR4 *ssrA::Km^r^*	This study
TIGR4*ΔsmpB*	TIGR4 *smpB::Km^r^*	Moreira et al., 2012
R6	Non-encapsulated strain derived from the capsular type 2 clinical isolate strain D39	Laboratory collection
R6*ΔssrA*	R6 *ssrA::Km^r^*	This study
R6*ΔssrA*(ROM)	R6*ΔssrA* [pROM]	This study
R6*ΔssrA*(*ssrA^+^*)	R6*ΔssrA* [pROM-TM]	This study
**Plasmids**		
pLS1ROM		Ruiz-Masó et al., 2012
pROM	pLS1ROM lacking PM promoter	This study
pROM-TM	pROM containing tmRNA chromosomal fragment	This study

**Table 2:** List of primers used in this study.

*Primer*	*Nucleotide sequence 5′–3′*	*Description*
tmRNA-F	TTCTGTGTCAGGGTAAGTTCC	Up PCR fragment for *ΔssrA* construction
tmRNAKmUp-R	TTATCCATTAAAAATCAAACGGATCACATACCTAAGATGAAGCTATCT	
tmRNA-R	ACTGAATCACCTCCTGTTATCG	Down PCR fragment for *ΔssrA* construction
tmRNAKmDown-F	TACGAGGAATTTGTATCGATGTGGACGTGGGTTCGACT	
KmN-F	CCGTTTGATTTTTAATGGATAA	Km^r^ cassette for *ΔssrA* construction
KmN-R	CATCGATACAAATTCCTCGTA	
pLSROM-F-Xba2	CGTCTGCAAAATACTCTAGAGATGGATCAAG	For pROM construction
pLSROM-R-Xba2	CTCACGAGACAGTCTAGAAAGTACAAAACCTCC	
tmRNACj-F	CGCGGATCCTTCATCTTAGGTATGTGATTTC	For *ssrA* cloning
tmRNACj-R	GCGCAAGCTTGGTCTGTTTGTGACTCCC	
tmRNA-1	AATTATCCTGCGCTCCAGAA	For *ΔssrA* construction and DNA sequencing
tmRNA-2	TTTCAAGACACGGCTGACA	

*Underlined sequence indicates enzyme restriction sites.*

### Construction of Genetically Modified Strains

**FIGURE 1:**
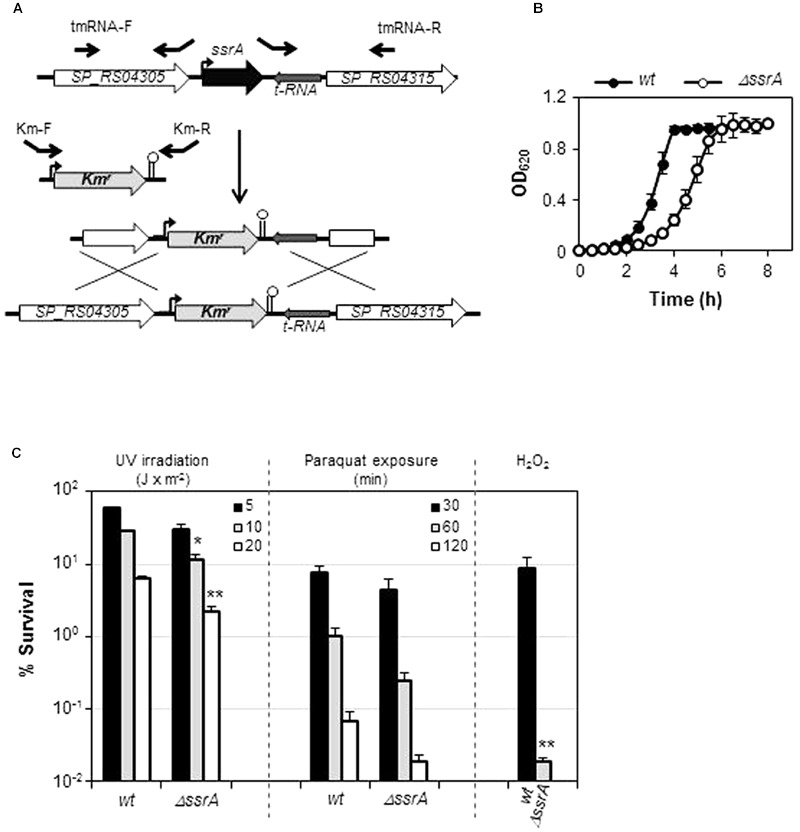
**Transfer messenger RNA (tmRNA) deficiency is detrimental under certain stress conditions. (A)** Inactivation of *ssrA* gene (black) by insertion/deletion of Km^r^ (light gray). Flanking ORFs (white) and tRNA gene (dark gray) are depicted. Promoters (curved arrows), terminators (stem-loop structures) and primers used (black arrows) are shown **(B)** Growth curves of wild type TIGR4 (*wt*) strain and its tmRNA deletion mutant (*ΔssrA*) in THY broth at 37°C. **(C)** Survival of *wt* and *ΔssrA* upon treatment with 30 mM paraquat at the indicated times, after UV irradiation at the intensities indicated, or after 30 min of exposure to 20 mM of hydrogen peroxide. Survival was determined as described in Section “Materials and Methods.” Values (mean ± SD) are the average of at least three independent experiments and the means were statistically compared using Student’s *t*-test (^∗^*P* < 0.05; ^∗∗^*P* < 0.01).

The TIGR4 tmRNA deletion mutant (TIGR4*ΔssrA*) was constructed by insertion-deletion of a kanamycin resistance cassette (Km^r^) through allelic replacement mutagenesis (Song et al., 2005). For this purpose, three fragments were generated by PCR. Two of them contained the upstream and downstream tmRNA regions and were obtained using oligonucleotide pairs tmRNA-F/tmRNAKmUp-R and tmRNAKmDown-F/tmRNA-R, respectively (**Table 2**). The third fragment, containing Km^r^, was amplified from pR410 plasmid (Sung et al., 2001) using KmN-F and KmN-R primers (**Table 2**). Primers tmRNAKmUp-R and tmRNAKmDown-F partially overlapped with KmN-F and KmN-R, respectively, to allow further hybridization of up- and downstream fragments with the amplified Km^r^ fragment. The three resulting PCR products were purified, mixed together, amplified using tmRNA-F and tmRNA-R primers, and used to transform strain TIGR4 (**Figure 1A**). Construction of the R6 tmRNA deletion strain (R6*ΔssrA*) was performed through amplification of a 3744 bp fragment encompassing tmRNA insertion/deletion from TIGR4*ΔssrA* chromosomal DNA using tmRNA-1 and tmRNA-2 primers (**Table 2**) and further transformation of R6. In both cases, transformants were selected with 250 μg/ml of Km, and confirmed by PCR amplification with external oligonucleotides tmRNA-1 and tmRNA-2 and DNA sequencing.

### Hydrogen Peroxidase and Paraquat Sensitivity Assays

Hydrogen peroxidase sensitivity assays were performed essentially as described by Pericone et al. (2003). Briefly, bacteria were grown in THY broth until an absorbance at 600 nm (*A_600_*) of 0.2 and diluted twofold in the same medium containing 40 mM H_2_O_2_ (Sigma-Aldrich), resulting in an true exposure of 20 mM H_2_O_2_. Bacteria were incubated at 37°C for 30 min and reactions were stopped by adding 200 U of bovine liver catalase (Sigma-Aldrich). Serial dilutions were plated in blood agar plates and incubated overnight at 37°C in a 5% CO_2_ atmosphere. Percentage of survival cells was calculated relative to the untreated control bacteria. Paraquat sensitivity assays were performed as previously described (Andisi et al., 2012). Bacteria were grown until *A_600_* = 0.2, and diluted twofold in the same medium with 30 mM paraquat (Sigma-Aldrich) or without paraquat, and incubated at 37°C for 2-h. Samples were taken at time intervals and the number of viable cells was determined by plating onto blood agar plates.

### Susceptibility to UV Irradiation Assays

Susceptibility to UV irradiation assays were performed as previously described (Halpern et al., 2004). Cells were grown in A+SY medium to exponential phase (an *A_620_* of approximately 0.2). Serial dilutions were then plated and exposed to UV light at 254 nm at the indicated intensities. Percentage of survival was estimated by colony counting relative to untreated control samples.

### Antibiotic Susceptibility Studies and Detection of Reactive Oxygen Species

MICs were determined by the broth macrodilution method (Clinical and Laboratory Standards Institute, 2008). To measure lethal action of antibiotics, pneumococci were grown in A+SY medium to *A_620_* = 0.4, diluted 100-fold and treated with the antibiotic. Samples were withdrawn at time intervals and colony formation was determined by plating on drug-free blood agar plates. The percentage of surviving cells was calculated relative to untreated control samples. When indicated, 10 μg/ml of chloramphenicol or 0.3 μg/ml of erythromycin was added to the culture 10 min prior to FQ addition and maintained throughout treatment. Intracellular oxidation level upon FQ treatment was measured by ROS detection using dihydrorhodamine 123 dye (Sigma-Aldrich) as previously described (Ferrándiz and de la Campa, 2014). Results were expressed as relative fluorescence units (RFU) and were made relative to time point zero, and normalized according to the number of live cells at each time point.

### Ethidium Bromide Uptake and Eﬄux Studies

Uptake of ethidium bromide (EtBr) was measured by fluorescence spectrophotometry as previously described (Pérez-Boto et al., 2015) with several modifications. Bacteria were grown at 37°C to mid-logarithmic phase (*A_620_* = 0.4) in A+SY medium. Cells were harvested, washed and suspended in phosphate saline buffer (PBS) (pH 7.2) with 0.2% glucose to *A_620_* = 0.2. After 10 min of incubation at 37°C, bacteria were exposed to 2 μg/ml of EtBr, with or without 20 μg/ml reserpine. Increase of fluorescence as EtBr entered the cells was directly recorded along 20 min, measuring every 0.25 s at 530 nm (excitation wavelength) and 600 nm (emission wavelength) in a Varian Cary Eclipse Spectrophotometer (Agilent Technologies Spain S. L. Madrid, Spain). The mean of at least three independent experiments was obtained.

Measurement of EtBr eﬄux was based on previously described method (Jumbe et al., 2006). Bacterial suspensions at *A_620_* = 0.2 were prepared in PBS with 0.2% glucose as described above. Bacteria were then exposed to EtBr for 20 min at 37°C in the presence of reserpine to maximize EtBr loading into bacteria. Cells were collected by centrifugation and suspended in fresh PBS + 0.2% glucose. Eﬄux of EtBr from cells was measured as fluorescence signal decrease during 20 min.

### FQ Accumulation Measurements

Accumulation of LVX on each strain was measured by fluorescence essentially as previously described (Piddock and Johnson, 2002). A starter culture was diluted 40-fold in 100 ml of A+SY medium, and grown at 37°C to *A_620_* = 0.5. Cells were washed and concentrated 20-fold in 0.1 M sodium phosphate pH 7.0, and the suspension was equilibrated for 10 min at 37°C prior to accumulation measurements. LVX was added at different concentrations and suspensions were incubated at 37°C for the indicated times. 0.5 ml aliquots were withdrawn and added to 2.5 ml of ice-cold 0.1 M sodium phosphate, washed with the same buffer and resuspended in 0.1 M glycine pH 3 to achieve cell lysis. After incubating at room temperature overnight, samples were centrifuged twice at 10000 ×*g* for 10 min. LVX concentration in the supernatant was measured by fluorescence spectroscopy at 295 nm (excitation wavelength) and 496 nm (emission wavelength), and compared to a standard fluorescence curve previously obtained. Accumulation data were converted into μg of LVX per ml.

### Analysis of Chromosome Fragmentation by PFGE

Chromosomal fragmentation was detected by pulsed-field gel electrophoresis (PFGE) as previously described (Ferrándiz et al., 2016). A starter culture was diluted 40-fold in A+SY medium and grown at 37°C to *A_620_* = 0.3. Bacteria were then treated with 10× MIC of LVX and incubated at 37°C for an additional 30 min. Cells were harvested, washed twice with wash buffer (1 M NaCl, 10 mM Tris pH 8) and inserted in solid agarose blocks for further lysis as described previously (Ferrándiz et al., 2016). Electrophoresis was performed in a Cheff-DR III system (Bio-Rad), for 20 h at 5.8 V/cm with a 0.1- to 40-s switch-time ramp at 14°C. Gels were stained with 0.5 μg/ml EtBr for 1 h and further distained in water during the same time. Percentages of chromosomal fragmentation were estimated by quantification of relative band intensities in each lane, corresponding to fragmented (compression zone) and non-fragmented (retained in the well) DNA.

## Results

### Deficiency of tmRNA Reduces Bacterial Growth and Increases Sensitivity to Stress

Transfer messenger RNA is a major actor in the *trans*-translation mechanism and, although its contribution to cell survival differs among bacteria, its inactivation is expected to diminish recovery from stress. To investigate the role of tmRNA in *S. pneumoniae* we constructed a TIGR4 tmRNA mutant (TIGR4*ΔssrA*) by deletion of its *ssrA* encoding gene and insertion of Km^r^ (**Figure 1A**). Deletion of *ssrA* increased the doubling time at 37°C from 42.8 ± 1.9 min (mean ± SD) to ∼61.7 ± 2.4 min (**Table 3**). These results indicated that, although tmRNA is not essential for the pneumococcus, its absence has a detrimental effect on growth (**Figure 1B**). We next evaluated the sensitivity to various environmental stress conditions. TIGR4*ΔssrA* was 3- to 4-fold more susceptible than TIGR4 to 30 mM paraquat, which induces formation of superoxide inside the cell (**Figure 1C**). However, these differences, although evident, were not statistically significant (*P* = 0.055 after 60 min and *P* = 0.092 after 120 min). By contrast, TIGR4*ΔssrA* was significantly more susceptible to treatment with UV light at 254 nm, with survival rates 2.5-fold (at 10 J × m^-2^; *P* = 0.015) and threefold (at 20 J × m^-2^; *P* = 0.0027) lower than TIGR4 (**Figure 1C**). Moreover, addition of exogenous H_2_O_2_ was highly deleterious for TIGR4*ΔssrA*, whose survival was reduced ∼400-fold after 30 min of incubation with 20 mM H_2_O_2_ (*P* = 0.005) (**Figure 1C**). These results revealed the importance of tmRNA and the *trans-*translation mechanism in adaptation of *S. pneumoniae* to certain environmental stress conditions.

**Table 3:** Doubling time of pneumococcal strains used.

*Strain*	*Duplication time (min)^1^*	
	37°C	30°C
TIGR4	42.8 ± 1.9	n.d.
TIGR4*ΔssrA*	61.7 ± 2.4	n.d.
R6	48.6 ± 0.9	65.4 ± 1.0
R6*ΔssrA*	62.2 ± 2.2	95.7 ± 4.5
R6*ΔssrA*(ROM)	85.4 ± 5.6	108.1 ± 10.1
R6*ΔssrA*(*ssrA^+^*)	41.0 ± 2.4	68.6 ± 1.5

*^1^Values were calculated from growth curves performed in A+SY medium at 37 or 30°C in a TECAN infinite F200 plate reader and are the average ± standard deviation of, at least, three independent experiments. n.d., not determined.*

We then examined the effect of the *ssrA* deletion under antimicrobial stress and determined the MIC values of several drugs from different families (**Table 4**). Deletion of *ssrA* lowered the MICs of antibiotics targeting translation, such as chloramphenicol (CM), erythromycin (EM) and tetracycline, and transcription, such as rifampicin, 2- or 4-fold, but had no effect with beta-lactams such as penicillin or cefotaxime, which interfere with cell wall synthesis. However, the MICs of MOX and LVX were higher for the *ΔssrA* strain than for the wild type by 1 dilution factor (**Table 4**). Such difference, although low, suggested that the tmRNA deletion reduces susceptibility to these drugs.

**Table 4:** *In vitro* antibiotic susceptibilities determined by macrodilution in TIGR4 and R6 strains of *Streptococcus pneumoniae*.

*Drug*	*MIC μg/mL^1^*				*Fold change^2^*
	TIGR4	TIGR4*ΔssrA*	R6	R6*ΔssrA*	
Chloramphenicol	1	0.5	n.d.	n.d.	2 ↓
Tetracycline	0.125	0.062	n.d.	n.d.	2 ↓
Erythromycin	0.03	0.0075	n.d.	n.d.	4 ↓
Rifampicin	0.06	0.03	n.d.	n.d.	2 ↓
Penicillin	0.016	0.016	n.d.	n.d.	
Cefotaxime	0.015	0.015	n.d.	n.d.	
Moxifloxacin	0.25	0.50	0.125	0.25	2 **↑**
Levofloxacin	0.50	1	0.25	0.50	2 **↑**
Ciprofloxacin	n.d.	n.d.	0.50	1	2 **↑**
Norfloxacin	n.d.	n.d.	4	8	2 **↑**

*^1^Results are the average of at least three independent replicates. n.d., not determined. ^2^Fold change denotes the ration between the values for the wild type strains and their corresponding *ΔssrA* derivative.*

### Absence of tmRNA Increases Resistance to FQs and Reduces ROS Production

**FIGURE 2:**
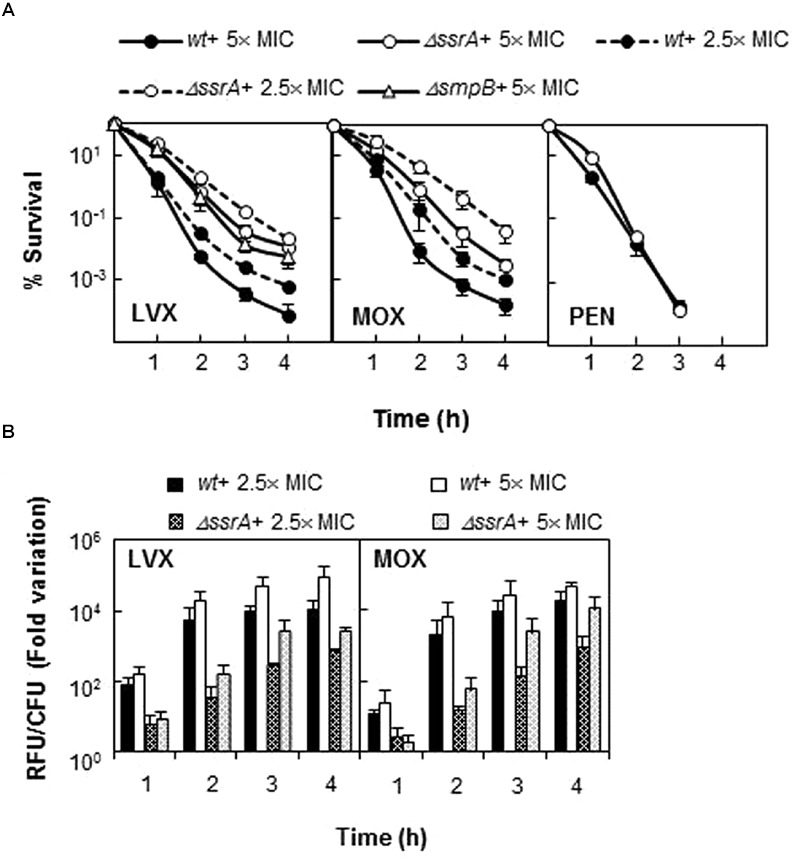
**Deletion of tmRNA reduces lethality of FQs but not of penicillin. (A)** Percentage of survival of *wt, ΔssrA* and TIGR4*ΔsmpB* (*ΔsmpB*) strains upon treatment with LVX, MOX and/or penicillin (PEN). Exponentially growing cells were treated with each antibiotic at the concentration and times indicated. Survival was determined as described in Section “Materials and Methods.” **(B)** Accumulation of ROS in *wt* and *ΔssrA* strains upon treatment with 2.5 and 5× MIC of LVX and MOX were measured in the same samples as described in Section “Materials and Methods.” RFU, relative fluorescence units; fluorescence units were divided by the number of viable cells and normalized to time zero and no antibiotic treatment condition. Values (mean ± SD) of at least three independent experiments are shown. The MIC values used were those determined in **Table 4**.

To confirm the apparent lower susceptibility of *ΔssrA* and better understand the effect of tmRNA on FQ activity, we determined the survival of wild type and mutant strains upon treatment with different concentrations of LVX and MOX from 1 to 4 h. The TIGR4*ΔssrA* was much more resistant to killing by both FQs at all times and concentrations tested, showing up to ∼150- (for LVX) and ∼100-fold (for MOX) higher survival rates than the wild type (**Figure 2A**). This effect was specific to FQs as no differences in survival were observed with the other bactericidal agent penicillin (**Figure 2A**). Moreover, this phenotype was associated to defects in *trans-*translation since similar survival rates to 5× MIC of LVX were observed with the *ΔsmpB* mutant lacking SmpB, the other indispensable partner for *trans-*translation (**Figure 2A**). Provided that FQ lethality is associated to ROS production we determined the internal levels of ROS in TIGR4 and TIGR4*ΔssrA* upon LVX and MOX treatment at the same time points and antibiotic concentrations. ROS accumulation in TIGR4*ΔssrA* was highly reduced compared to TIGR4, showing a decrease of more than ∼120-fold after 2 h treatment with both antibiotics (**Figure 2B**). These results demonstrated that the higher survival rate exhibited by TIGR4*ΔssrA* is related with a reduction in ROS production.

**FIGURE 3:**
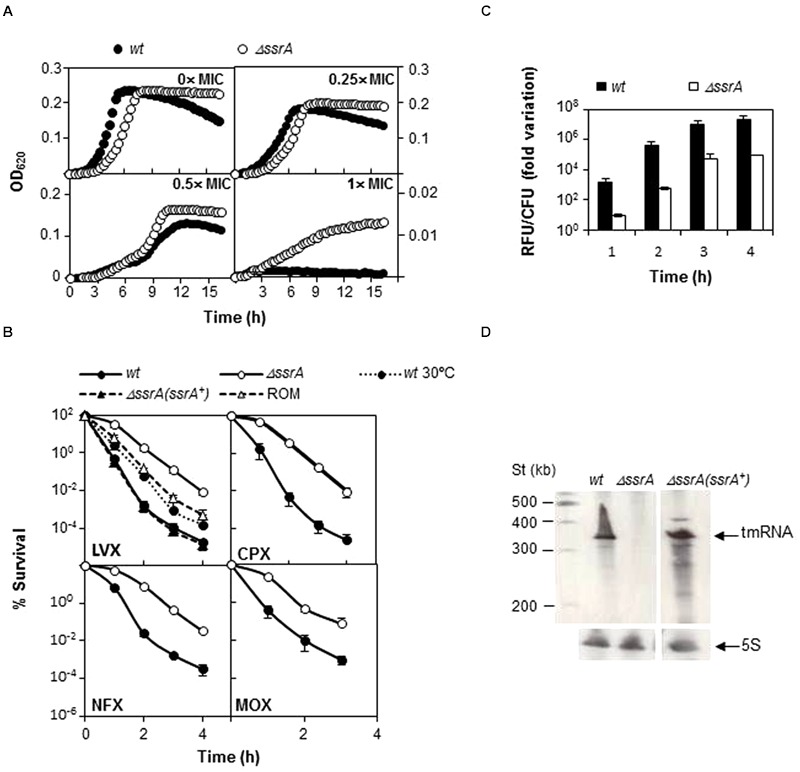
**Transfer messenger RNA deficiency protects from FQs lethal action. (A)** Wild type R6 strain (*wt*) and its tmRNA deletion mutant (*ΔssrA*) were grown under different LVX concentrations in A+Y medium. Growth was followed by turbidity (OD_620__nm_) in a TECAN infinite F200 plate reader at 37°C. **(B)** Percentage of survival to different FQs of *wt, ΔssrA*, the complemented strain containing the pROM-TM (*ΔssrA*(*ssrA^+^*)) and the *ΔssrA* containing the empty vector (ROM) at 37°C, and of *wt* at 30°C. Exponentially growing cells were treated with 5× MIC (as determined in **Table 4**) of LVX, ciprofloxacin (CPX), norfloxacin (NFX), or MOX for the times indicated. After incubation, survival was determined as described in Section “Materials and Methods.” **(C)** Accumulation of ROS in *wt* and *ΔssrA* upon addition of 5× MIC of LVX was measured as described in Section “Materials and Methods.” RFU; fluorescence units were divided by the number of viable cells and normalized to time zero and no antibiotic treatment condition. **(D)** Northern-blot showing tmRNA expression in *wt, ΔssrA* and *ΔssrA(ssrA^+^*). Bands corresponding to tmRNA and the control 5S rRNA are indicated by arrows. Northern-blots were performed as described (Acebo et al., 2012). Values (mean ± SD) of at least three independent experiments are shown. MIC values were determined in **Table 4**.

To ensure that the less FQ-sensitive phenotype of the tmRNA deficiency was not related with the genetic background, a similar analysis was performed using strain R6. We constructed the R6*ΔssrA* strain and analyzed growth under different concentrations of LVX. As shown in **Figure 3A**, growth inhibition upon antibiotic treatment was lower in R6*ΔssrA* than in R6, and the differences between strains increased proportionally with LVX concentration up to 1 μg/ml. The study was extended to other FQs and the MIC values of LVX, MOX, ciprofloxacin (CPX), and norfloxacin (NFX) were determined. In all cases, the MICs were higher for R6*ΔssrA* than for R6 (**Table 4**), confirming less susceptibility to all FQs. We then compared survival of R6 and R6*ΔssrA* strains upon treatment with the four distinct FQs (**Figure 3B**). As in the TIGR4 genetic background, the lethal action of the four FQs was reduced several 100-fold in the R6*ΔssrA*, and this effect was associated with >2-log decrease in ROS accumulation after LVX treatment (**Figure 3C**). To attribute protection from FQ lethal activity to tmRNA deficiency, complementation experiments were performed. The pROM-TM plasmid containing the entire *ssrA* gene was constructed and introduced into R6*ΔssrA*. Expression of tmRNA in *trans* in this strain was confirmed by Northern blot-analysis (**Figure 3D**). Survival during LVX treatment revealed that the complemented strain was killed to the same extent as R6, while R6*ΔssrA* with the empty vector did not (**Figure 3B**). Furthermore, since the potency of bactericidal compounds may increase with faster bacterial growth rate (Deitz et al., 1966), the possibility exists that the higher survival to FQs shown by R6*ΔssrA* resulted from its slower growth. To exclude this possibility, we analyzed survival of R6 to LVX at 30°C, which reduced the doubling time of wild type cells (65.4 ± 1.0 min) to the same levels as the R6*ΔssrA* mutant at 37°C (62.2 ± 2.2 min) (**Table 3**). Results showed that resistance to LVX of R6 indeed partially increased at 30°C, showing 7.7- and 8.4-fold higher percentages of survival than at 37°C after 3 and 4 h treatment, respectively (**Figure 3B**). However, these values were far from those obtained with R6*ΔssrA* at 37°C, which showed survival percentages of about 1100- and 450-fold higher after 3 and 4 h of LVX-treatment, respectively.

From these experiments we can conclude that *ΔssrA* strain is better able to survive to the lethal effect of FQs than *ssrA^+^* cells and that this phenotype does not relay only in growth defect, but in another mechanism of protection directly linked with defects in *trans-*translation.

### tmRNA Deficiency Increases FQ Accumulation

**FIGURE 4:**
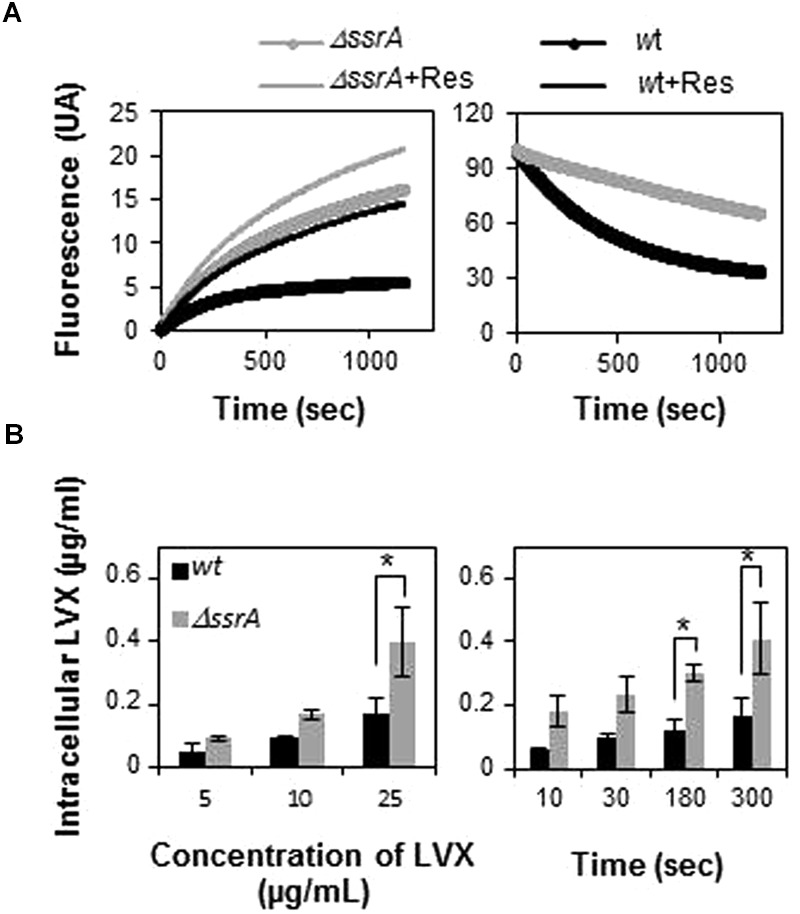
**Transfer messenger RNA deficiency leads to higher intracellular accumulation of drugs. (A)** EtBr uptake (left panel) and eﬄux (right panel) of R6 (*wt*) and R6*ΔssrA* (*ΔssrA*) strains during 20 min was measured as described in Section “Materials and Methods.” Fluorescence intensity represents levels of intracellular EtBr. The change in fluorescence is normalized to the initial levels to allow for direct comparison. Fluorescence was directly recorded during 20 min after addition of EtBr in the presence (+Res) or in the absence of reserpine. **(B)** Fluorometric measurement of intracellular accumulation of levofloxacin (LVX) in *wt* and *ΔssrA* at different LVX concentrations (left panel) and time points (right panel), as described in Section “Materials and Methods.” Three to five independent experiments were conducted and the mean values were statistically compared using Student’s *t*-test (^∗^*P* < 0.05).

The less sensitive phenotype of the tmRNA deletion mutant could be due to a lesser accumulation of FQs inside the cell, due to changes in membrane permeability. To explore this possibility, we conducted experiments comparing EtBr uptake and eﬄux in both R6 and R6*ΔssrA* strains. Results revealed that EtBr uptake was higher in R6*ΔssrA* than in R6 and, simultaneously, the eﬄux was lower (**Figure 4A**). As a consequence, internal levels of EtBr in R6*ΔssrA* were higher than in R6. Addition of reserpine, an eﬄux pump inhibitor in streptococci (Ferrándiz et al., 1999; Piddock and Johnson, 2002), considerably increased EtBr accumulation in the wild type strain, while the effect in R6*ΔssrA* was much lower. Remarkably, EtBr levels accumulated in R6 upon reserpine addition were equivalent to the levels in R6*ΔssrA* without the inhibitor. These results indicate that the eﬄux pumps in R6*ΔssrA* are less active in exporting EtBr, leading to higher accumulation of this drug. We analyzed whether the observed effect was specific to EtBr or whether the accumulation of LVX was also increased in the *ΔssrA* mutant. We examined levels of intracellular LVX upon treatment with different antibiotic concentrations and different exposure times. Again, R6*ΔssrA* accumulated higher amounts of LVX than R6 in all cases (**Figure 4B**). These results suggest that the absence of tmRNA indeed induces changes in *S. pneumoniae* membrane permeability. However, instead of reducing levels of EtBr and LVX inside the cell, these changes lead to their higher accumulation.

### Absence of tmRNA Protects from Chromosomal Fragmentation Associated to FQs

**FIGURE 5:**
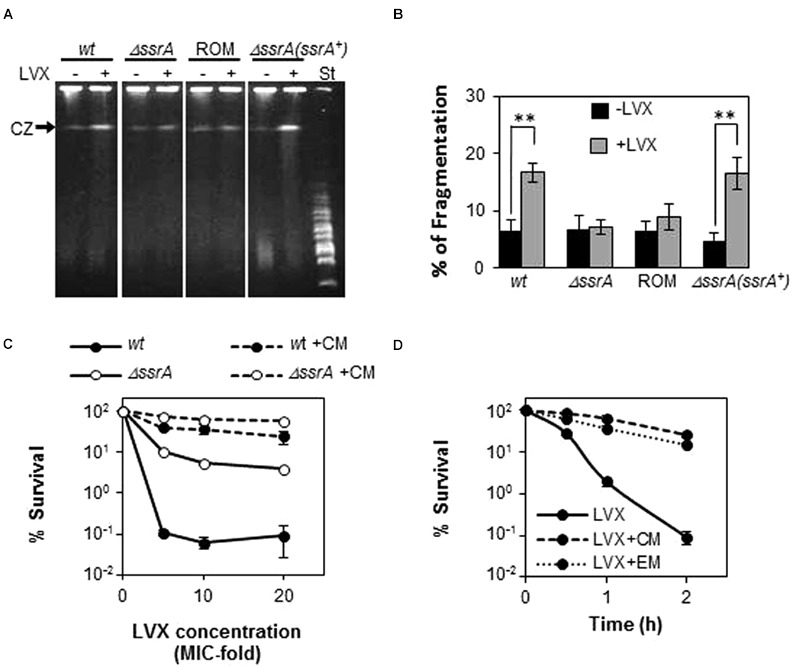
**Deficiency of tmRNA protects from chromosome fragmentation mediated by LVX. (A)** Analysis of chromosome fragmentation upon LVX treatment by PFGE. Exponentially growing cultures of R6 (*wt*), the R6*ΔssrA* (*ΔssrA*) mutant, the complemented strain containing the pROM-TM (*ΔssrA(ssrA^+^)*) and the R6*ΔssrA* containing the empty vector (ROM), were treated with or without 10× MIC of LVX for 30 min and analyzed by PFGE as described in Section “Materials and Methods.” **(B)** Chromosomal fragmentation was estimated by quantification of the compression zone (CZ) relative to the intact chromosomal DNA retained in the well. Each value is the mean of at least three independent experiments and the mean values were statistically compared using Student’s *t*-test (^∗∗^*P* < 0.01). **(C)** Percentage of survival to 1 h treatment of LVX at 5, 10, or 20× MIC of *wt, ΔssrA* strains previously incubated with 10 μ/ml of Chloramphenicol (CM) for 10 min. St stands for the MidRange PFG Marker (Biolabs) with size ranges from 15 to 300 Kb. **(D)** Percentage of survival along the time to treatment with 5× MIC of *wt* strains previously incubated with 10 μ/ml of CM or 0.3 μ/ml of erythromycin (EM) for 10 min. Survival was determined as described in Section “Materials and Methods.”

Since the lethal effect of FQs has been correlated with chromosome fragmentation, we examined LVX-associated DNA breakage in R6 and R6*ΔssrA* after 30 min of exposure to 10× MIC of LVX (**Figure 5**). Percentage of the bands corresponding to the compression zone (CZ, containing the large size nicked fragments of chromosomal DNA) was used to estimate chromosomal fragmentation. As shown in **Figures 5A,B**, LVX treatment induced a 16.7 ± 1.6% (mean ± SD) fragmentation in R6, compared with the 6.5 ± 1.9% observed without the antibiotic. However, such increase was not observed in R6*ΔssrA*, whose fragmentation levels with and without LVX were similar (7.1 ± 1.25 and 6.7 ± 2.6%, respectively). Interestingly, overexpression of tmRNA in the complemented strain completely restored the initial levels of fragmentation to 16.5 ± 2.7%, while the empty vector did not (8.8 ± 2.2%). Therefore, we can conclude that the absence of tmRNA protects from chromosome fragmentation associated to FQs treatment.

Absence of tmRNA is expected to slow down translation due to lack of turnover of stalled ribosomes and it is known that in *Escherichia coli* several FQs require active protein synthesis to cause cell death (Malik et al., 2006, 2007). Therefore, it is possible that the protective effect of *ΔssrA* could be related to a lower protein synthesis rate. To explore this possibility, we tested the effect of the protein inhibitor CM as a blocking agent of FQ-lethal action in the pneumococcus. For this purpose, bacterial cultures were treated with 10× MIC of CM for 10 min, and then different concentrations of LVX were added for 1 additional hour. As shown in **Figure 5C**, incubation with CM drastically reduced cell death in R6, increasing survival from ∼0.1% in the absence of the protein inhibitor, to values ranging between 25 and 60%. Similar percentages of survival were observed in R6*ΔssrA* when treated with CM, in which cell death levels without protein inhibitor treatment were also remarkably higher (5–10%) than in the wild type. The protective effect of CM increased at longer times and it was extended to other protein inhibitors such as EM. Incubation with both antibiotics increased survival to LVX after 2 h-treatment more than 200-fold (**Figure 5D**). This means that LVX lethal action in *S. pneumoniae* also requires active protein synthesis, what could explain the less susceptible phenotype of *ΔssrA* cells.

## Discussion

In this study we provide evidence that *S. pneumoniae* relies on tmRNA for adaptation to a variety of environmental conditions, and that its inactivation reduces the ability of the pneumococcus to cope with several stresses. Our findings showed that tmRNA is not essential for growth in the pneumococcus, although its absence significantly reduced growth rate. Most bacteria require a system to resolve non-stop complexes (Keiler and Feaga, 2014). On this regard, *S. pneumoniae* is not an exception. The presence of a putative ArfB encoding gene has been predicted in the pneumococcal genome (Keiler and Feaga, 2014) and this is probably the reason why tmRNA is not essential for growth. Moreover, we demonstrated that insertional inactivation of tmRNA did not affect survival to internal oxidative stress induced by paraquat, but reduced the ability to survive to the lethal effect of UV irradiation and had a deleterious effect upon exposure to exogenous H_2_O_2_. We also demonstrated that pneumococcal cells lacking tmRNA exhibited higher susceptibility to antibiotics that inhibit protein synthesis or transcription, but not to those targeting cell wall synthesis. The increased sensitivity to protein synthesis inhibitors was rather moderate, similarly to what was previously observed for *E. coli* (Abo et al., 2002), *Synechocystis* (de la Cruz and Vioque, 2001) or *Francisella tularensis* (Svetlanov et al., 2012), whose *ssrA* null mutants were more sensitive to sublethal concentrations of these drugs. Inhibition of the translation elongation process may cause ribosome stalling, translational inaccuracy and/or read-through, leading to the accumulation of non-stop complexes (Thompson et al., 2002, 2004; Vioque and de la Cruz, 2003). Therefore, the *trans-*translation system confers the cells with partial resistance to certain antibiotics by dealing with ribosome pausing. In case of rifampicin, the increment in sensitivity exhibited by the *ΔssrA* mutant was also moderate. A similar sensitive phenotype was observed in *F. tularensis* (Svetlanov et al., 2012) but no in *E. coli* or *Synechocystis* (de la Cruz and Vioque, 2001; Abo et al., 2002), suggesting certain differences among bacteria. This antibiotic inhibits transcription and no ribosome stalling is expected upon rifampicin treatment. However, translation and transcription are known to be coupled and defects in RNA synthesis may ultimately have consequences in translation.

Remarkably, our findings demonstrated that pneumococcal cells lacking tmRNA were more resistant to the four FQs tested (LVX, MOX, CPX, and NFX), showing both higher MIC values (twofold) and higher survival rates (up to 1100-fold). Such resistant phenotype was not observed with penicillin and was not due to the lower growth rate shown by the tmRNA deletion mutant, since increasing the doubling time of wild type at 30°C had no the same effect. Moreover, this protective phenotype was associated to *trans-*translation defects and not to side effects of the tmRNA, since similar survival rates were observed in a *ΔsmpB* strain. This is the first study reporting a higher FQ-resistant phenotype associated to tmRNA inactivation. These results contrast with those previously obtained in *E. coli* or *F. tularensis*, where absence of tmRNA was previously reported to either have no effect (Abo et al., 2000; Luidalepp et al., 2005; Svetlanov et al., 2012) or to increase sensitivity (Nichols et al., 2011; Li et al., 2013) to nalidixic acid, ofloxacin, NFX or LVX. A possible explanation for the less sensitive phenotype observed in *S. pneumoniae* could be that depletion of tmRNA might influence membrane permeability, leading to lesser accumulation of the drug inside *ΔssrA* cells. In fact, absence of tmRNA is expected to prevents tmRNA-mediated tagging of abnormal proteins for degradation and to increases the levels of misfolded proteins (Gottesman et al., 1998; Herman et al., 1998; Choy et al., 2007), whose insertion into cell membrane could alter permeability. However, our results demonstrated that cells lacking tmRNA accumulated more EtBr and LVX than the wild type cells. Therefore, cell membrane integrity was indeed altered due to the deletion, but instead of reducing drug accumulation, it led to a significant increase in the levels of intracellular drugs. This means that tmRNA-depleted cells are somehow protected from the lethal effects associated to FQs.

ROS production contributes to FQ-lethality in *S. pneumoniae*. The intervening pathways between LVX and MOX initial antibiotic-target interaction and ROS formation are now known. LVX treatment inhibits Topo IV increasing *fatDCE* operon expression, what in turns, increases iron uptake (Ferrándiz and de la Campa, 2014). In the case of MOX, inhibition of both Topo IV and gyrase induce a transcriptomic response that results in an intracellular pyruvate increment that consequently leads to higher levels of H_2_O_2_ (Ferrándiz et al., 2016). Consistently, our results demonstrated that the protective effect against LVX and MOX of *ΔssrA* cells was accompanied by a reduction in ROS accumulation. A similar protective effect was also observed against NFX and CPX, both of which target Topo IV (Muñoz and De La Campa, 1996), and whose induction of intracellular ROS in *S. pneumoniae* has not been determined. In *E. coli*, these FQs have been shown to act through different pathways, and while NFX-mediated killing involves ROS (Dwyer et al., 2007; Kohanski et al., 2007), CPX is able to kill bacteria anaerobically (Malik et al., 2007). Therefore, the protective effect of tmRNA absence may not be related to specific pathways for each FQ, but to a common feature to all of them. Nevertheless, since MOX addition in pneumococcus triggered a threefold reduction in *smpB* transcript levels (Ferrándiz et al., 2016), the involvement of tmRNA in specific regulatory circuits contributing to FQ lethality cannot be ruled out.

Independently of their target and mechanism of action, FQs reversibly trap the topoisomerase on the chromosomal DNA, in a ternary complex in which the DNA is broken (Chen et al., 1996; Malik et al., 2006). Releasing of DNA from these complexes generates deleterious double-stranded breaks (Drlica et al., 2008). Therefore, no matter the pathway used, the action of all FQs ultimately converges in lethal chromosome fragmentation. On this regard, our findings demonstrated that cells lacking tmRNA showed almost no chromosome fragmentation after LVX treatment, and that the sole tmRNA expression in *trans* is sufficient to completely restore DNA fragmentation to wild type levels. We also demonstrated that addition of CM or EM, which inhibit protein synthesis, drastically increased survival of pneumococci exposed to LVX. These findings confirm that LVX-mediated cell death in pneumococcus also occurs via the protein synthesis-dependent pathway and provide a possible explanation to the less susceptible phenotype of *ΔssrA* cells. The absence of tmRNA is expected to imperil translation due to accumulation of stalled ribosomes in tmRNA depleted cells, which may give cells time to repair damage.

## Conclusion

Transfer messenger RNA has a protective effect under several types of stresses in *S. pneumoniae*. Such protection is likely due to its role in rescuing of stalled ribosomes, helping the cell to recover and proceed with proper translation, and to tagging of abnormal proteins for degradation. However, upon FQ treatment, tmRNA is paradoxically harmful. The protective effect against FQs associated to tmRNA deficiency is linked to *trans-*translation and is due to the cumulative effect of two processes: a reduction in ROS production and a decrease in chromosome fragmentation, and translational pausing appear to be the underlying mechanism. These findings should be taking in consideration in the development of new antibiotics inhibiting *trans-*translation. Its requirement for viability or virulence in many pathogenic bacteria together with the low expected toxicity in host cells due to its absence in metazoans, has posed this pathway as an attractive antimicrobial target (Keiler and Alumasa, 2013; Ramadoss et al., 2013; Keiler and Feaga, 2014). However, our results evidence that inactivation of *trans-*translation may have side effects leading to an undesirable increase resistance to otherwise effective antibiotics. In addition, although not currently used in clinical practice, our results suggest that the combined use of FQs and antibiotics inhibiting protein synthesis might not be recommended and evidence the requirement of further studies on this matter.

## Author Contributions

LB, JW, MF, AG-S, and MA performed the experimental work. MA, AC, and MF participated in study conception, data interpretation, and manuscript writing. All authors participated in manuscript corrections.

## Conflict of Interest Statement

The authors declare that the research was conducted in the absence of any commercial or financial relationships that could be construed as a potential conflict of interest.

## Footnotes

1 http://www.who.int
